# Tumor epitope spreading by a novel multivalent therapeutic cellular vaccine targeting cancer antigens to invariant NKT-triggered dendritic cells *in situ*

**DOI:** 10.3389/fimmu.2024.1345037

**Published:** 2024-02-01

**Authors:** Satoru Yamasaki, Kanako Shimizu, Shin-ichiro Fujii

**Affiliations:** ^1^Laboratory for Immunotherapy, RIKEN Research Center for Integrative Medical Science (IMS), Yokohama, Japan; ^2^aAVC Drug Translational Unit, RIKEN Center for Integrative Medical Science (IMS), Yokohama, Japan; ^3^RIKEN Program for Drug Discovery and Medical Technology Platforms, Yokohama, Japan

**Keywords:** cancer, cytotoxic T cell, dendritic cell, immunotherapy, iNKT cell

## Abstract

**Introduction:**

Cancer is categorized into two types based on the microenvironment: cold and hot tumors. The former is challenging to stimulate through immunity. The immunogenicity of cancer relies on the quality and quantity of cancer antigens, whether recognized by T cells or not. Successful cancer immunotherapy hinges on the cancer cell type, antigenicity and subsequent immune reactions. The T cell response is particularly crucial for secondary epitope spreading, although the factors affecting these mechanisms remain unknown. Prostate cancer often becomes resistant to standard therapy despite identifying several antigens, placing it among immunologically cold tumors. We aim to leverage prostate cancer antigens to investigate the potential induction of epitope spreading in cold tumors. This study specifically focuses on identifying factors involved in secondary epitope spreading based on artificial adjuvant vector cell (aAVC) therapy, a method established as invariant natural killer T (iNKT) -licensed DC therapy.

**Methods:**

We concentrated on three prostate cancer antigens (prostate-specific membrane antigen (PSMA), prostate-specific antigen (PSA), and prostatic acid phosphatase (PAP)). By introducing allogeneic cells with the antigen and murine CD1d mRNA, followed by α-galactosylceramide (α-GalCer) loading, we generated five types of aAVCs, i.e, monovalent, divalent and trivalent antigen-expressing aAVCs and four types of prostate antigen-expressing cold tumors. We evaluated iNKT activation and antigen-specific CD8+ T cell responses against tumor cells prompted by the aAVCs.

**Results:**

Our study revealed that monovalent aAVCs, expressing a single prostate antigen, primed T cells for primary tumor antigens and also induced T cells targeting additional tumor antigens by triggering a tumor antigen-spreading response. When we investigated the immune response by trivalent aAVC (aAVC-PROS), aAVC-PROS therapy elicited multiple antigen-specific CD8+ T cells simultaneously. These CD8+ T cells exhibited both preventive and therapeutic effects against tumor progression.

**Conclusions:**

The findings from this study highlight the promising role of tumor antigen-expressing aAVCs, in inducing efficient epitope spreading and generating robust immune responses against cancer. Our results also propose that multivalent antigen-expressing aAVCs present a promising therapeutic option and could be a more comprehensive therapy for treating cold tumors like prostate cancer.

## 1 Introduction

Prostate cancer (PCa) is considered a “cold tumor,” characterized by low immune cell infiltration, an immunosuppressive tumor immune microenvironment (TIME), and infiltrating effector lymphocytes with a dysfunctional phenotype (1). However, recent single-cell analyses have allowed stratification, and immunotherapy is effective in some cases. Clinically, PCa is the second most prevalent cancer in men and the fifth most common cause of cancer-related deaths worldwide (2). Prostatectomy, radiation therapy, chemotherapy, and hormone deprivation therapy have been used as traditional treatments for prostate cancer (3). However, these treatments have limited efficacy; thus, new approaches for combating prostate cancer are warranted (4). One of the reasons for the limited treatment options relies on the host’s immune dysfunction in innate and adaptive immunity. By investigating prostate cancer as an example, the clinical concerns of cold tumors can be analogized.

PCa stands as a significant example among cancers recognized for their diminished innate immunity. Patients with advanced PCa exhibit a decreased number of peripheral blood iNKT cells. Patients with aggressive stages of the disease had fewer iNKT cells (5). In considering the functional restoration of innate immunity, the advantage of the antitumor effect mediated by iNKT cells extends beyond the direct antitumor effect. It also includes the adjuvant effect on dendritic cells and the adjunct effect on natural killer (NK) cells. The adjunct effect on NK cells, wherein iNKT cells activate NK cells even in the presence of tumors, has been well-known because the validation of NK activation is an important indicator. Indeed, NK cells are major effector cells in cancer immunotherapy (6, 7). NK cell function is often inhibited at multiple levels in the PCa TME (8). Decreased or impaired NK activity has been reported as cancer progresses (9), and the frequency of circulating NK cells is reduced in metastatic PCa (10). Further, higher intratumoral NK cell numbers have been shown to confer longer distant metastasis-free survival (11). Thus, iNKT and NK cells, as innate lymphocytes, play a protective role against the aggressive state or metastasis of prostate cancer. PCa is a cancer known to have reduced T-cell immunity. Important evidence has emerged from clinical studies regarding the role of T cells in prostate cancer. Patients with PCa show limited responses to immune checkpoint inhibitors, which have revolutionized the treatment of several cancer types (12–15). This poor response to immune checkpoint blockade (ICB) treatment is attributed to the low infiltration of CD8^+^ T cells into prostate tumors compared to other solid tumors that are more responsive to checkpoint blockade (16, 17). Despite this, prostate cancer cells typically express several prostate-specific antigens, such as prostatic acid phosphatase (PAP), prostate-specific antigen (PSA), and prostate-specific membrane antigen (PSMA), which have been used as vaccine targets (18). Therefore, developing a strategy for immunotherapy against prostate cancer requires the expansion of prostate tumor antigen-specific CD8^+^ T cells and their trafficking into tumor tissues.

There are several cancers, such as PCa, pancreatic, ovarian and breast cancers, in which both NK and T cells are dysfunctional from a clinical standpoint (19). Immunological strategies that boost both should be considered for such cold tumors. Simultaneously generating NK cell and CD8^+^ T cell responses is ideal for preventing tumor cells from evading immune surveillance. Previous animal and clinical studies have reported on α-galactosylceramide (α-GalCer)-pulsed dendritic cell (DC) therapy. iNKT cells produce systemic IFN-γ responses, leading to NK cell-mediated antitumor effects (20–25). Recently, we reported that the number of granzyme B-expressing NK cells increases in the early stages of lung cancer (26). However, therapies focused solely on NK cell activation exhibit limited efficacy. We developed a cellular cancer vaccine termed artificial adjuvant vector cells (aAVCs) (27–29), based on the relationship between innate immunity and the induction of adaptive immunity via *in vivo* DC targeting (30, 31). These cells are allogeneic cells (NIH3T3 for murine and HEK293 for human) co-transfected with an antigen-derived mRNA and CD1d mRNA and then loaded with iNKT cell ligand. aAVC can activate iNKT and NK cells as an adjunct activity by iNKT cells. In a series of immune responses, after activation by aAVC, innate iNKT/NK cells reject the aAVCs, and then, the killed aAVCs are taken up by DCs *in situ*, thereby promoting several immunogenic features of DCs. The *in vivo* DCs in the lungs, liver, and spleen that captured aAVCs undergo maturation via interaction with iNKT cells, which is brought about by CD40L/CD40 interactions and inflammatory cytokines (IFN-γ and TNF-α) (27–29, 32, 33). aAVC therapy is a cellular therapy platform, and we demonstrated that many types of antigens were used, such as OVA, MART-1, NY-ESO-1, and WT1 antigens in murine models (27–29, 34–36) and also against human relapsed and refractory acute myelogenous leukemia patients (37). Such *in vivo* DC-targeting therapies promote CD8^+^ T cell responses more effectively than ex vivo DC therapies (28, 32, 33, 38).

This study has two primary aims. First, we attempted to establish an aAVC containing the cancer antigens and demonstrate an antitumor effect against the relevant antigen-expressing, cold tumor cells. We investigated whether aAVCs expressing a single antigen can induce tumor antigen-specific responses and demonstrate antigen spreading. Furthermore, we developed aAVCs containing two or three cancer antigens (PSMA, PSA, and PAP) aiming to induce multiple types of CD8^+^ T cells, thereby preventing tumor cells from evading immune detection. We examined how aAVCs, which express several antigens, activate multiple antigen-specific CD8^+^ T cells *in vivo*. We then compared the condition because the strength of the CD8^+^ T cell response depends on the antigenicity of the tumor antigen. Our findings demonstrate that multiple prostate-cancer antigen-expressing aAVCs can promote tumor epitope spreading as well as activate multiple antigen-specific CD8^+^ T cells, representing a promising therapeutic option for conventional therapy-resistant prostate cancer. Hence, restoring and activating innate immunity would lead to useful immunotherapy.

## 2 Materials and methods

### 2.1 Animals and cell lines

Pathogen-free C57BL/6 (B6) mice were purchased from Charles River at 6 to 8 weeks of age. Rag1 ^-/-^ (B6.129S7-*Rag1^tm1Mom^*/J) mice were purchased from the Jackson Laboratory. CD1d^-/-^ mice with B6 genetic background were kindly provided from L. Van Kaer (Vanderbilt University, Nashville, TN). Ly5.1 congenic OT-1 mice were generated by cross/backcross breeding of OT-1 with B6. Ly5.1 mice. All mice were maintained under specific pathogen-free conditions at the RIKEN animal facility, and all procedures were performed in compliance with the protocols approved by the Institutional Animal Care Committee at RIKEN (AEY2022-020). NIH3T3 cells were obtained from the RIKEN Cell Bank. NKT hybridoma (1.B2) and B16 cell line were kindly provided by Dr. M. Kronenberg (La Jolla Institute) and Dr. RM Steinman (Rockefeller University) respectively. All cell lines were maintained and treated according to the supplier’s recommendations. B16-PSMA, B16-PSA, and B16-PAP cells were generated by retroviral transduction of human PSMA, PSA, or PAP genes into B16 cells.

### 2.2 Reagents

#### 2.2.1 Antibodies, peptides and proteins

The following monoclonal antibody (mAbs) were purchased from BD Biosciences, or BioLegend: anti-CD1d (1B1), anti-CD3 (145-2C11), anti-CD8 (53-6.7), CD19(6D5), CD45.1 (A20), TCRβ (H57-597), Vα2/TCRVα2 (B20.1), TNF-α (MP6-XT22) and IFN-γ (XMG1.2) and CD1d-DimerX. The OVA-tetramer and CD1d-tetramer were purchased from MBL. Anti-PSMA (clone: UMAB27), anti-PSA (clone: EP1588Y), and anti-PAP (clone: EPR4067) antibodies were obtained from OriGene. OVA257–264 peptide (SIINFEKL) and α-GalCer were purchased from Toray Research Center, Inc., and Funakoshi, respectively. PepTivator PSMA (#130-099-795), PepTivator PSA (#130-099-800), and PepTivator PAP (#130-096-767) were purchased from Miltenyi Biotech Inc. The recombinant human PSMA (FOLH1), human PSA (KLK3), and human PAP (ACPP) proteins were purchased from Origene (Rockville, MD, USA).

#### 2.2.2 Cell preparation

Mononuclear cells (MNCs) from the spleen and liver were isolated as previously described. Briefly, spleen cells were obtained by passing the spleen through a 70 μm cell strainer. Subsequently, erythrocytes were lysed with ACK lysing buffer (Life Technologies). For the isolation of liver MNCs, the minced liver tissues were digested with collagenase D (Roche) and layered on Percoll gradients (40/70%) (Amersham Pharmacia Biotec), followed by centrifugation.

### 2.3 Flow cytometry

The spleen cells were preincubated with anti-CD16/CD32 (BioLegend) in PBS with 2% heat-inactivated FBS and then stained with surface antibodies and viability dye for 30 min on ice. A Fixable Aqua or Violet-Dead Cell Stain Kit (Invitrogen) was used to remove the dead cells. The cytokine production was analyzed by intracellular staining using a BD Intracellular Cytokine Staining Kit (BD Biosciences). For these, LSRFortessa X-20 or Canto II instrument and CELLQuest, FACSDiva (BD Biosciences), or the FlowJo software (v10.3B2) were used.

### 2.4 Preparation of aAVC-OVA, PSMA, aAVC-PSA, and aAVC-PAP

Murine CD1d and OVA plasmids used in this study was previously described (29). Coding sequences for PSMA (GenBank Accession Number: NM_004476), PSA (GenBank Accession Number: NM_001648), and PAP (GenBank Accession Number: NM_001099) were generated via gene synthesis (Takara) and cloned into the HindIII and BamHI, HindIII and EcoRI, and HindIII and BamHI sites of the pGEM-4Z vector (Promega), respectively. The resultant plasmid was then linearized with BamHI and EcoRI and purified using the QIAquick PCR Purification Kit (Qiagen). *In vitro* transcription was conducted using the mMessage mMachine T7 Ultra Kit (Ambion), and then RNA was purified using the RNeasy Mini/Midi Kit (Qiagen). aAVCs were prepared as previously described (27). Briefly, NIH3T3 cells resuspended in OptiMEM and RNA were transferred simultaneously to a cuvette. This cell suspension was pulsed in an ECM 830 Square Wave Electroporation System (Harvard Apparatus). After electroporation, the cells were transferred to a culture medium and cultured in 500 ng/mL of α-GalCer. Protein expression in the transfected cells was assessed by flow cytometry for CD1d, ELISA (ITEA) for OVA and western blot analysis for antigen proteins, such as PSMA, PSA and PAP.

### 2.5 Western blotting

To quantify the protein levels of prostate antigens (PSMA, PSA, and PAP), western blotting was used as previously described (35). Briefly, the relevant antigen-expressing aAVC was lysed in Laemmli sample buffer (Bio-Rad) and boiled at 95°C. Samples were loaded onto a polyacrylamide gel and electrotransferred to a PVDF membrane. Membranes were blocked and incubated with primary anti-PSMA, anti-PSA, or anti-PAP antibodies overnight at 4°C with gentle shaking. The membranes were incubated with horseradish peroxidase (HRP)-conjugated goat anti-mouse antibody (R&D Systems) at room temperature. Chemiluminescence images were obtained using a luminescence image analyzer (LAS 1000) and Image Gauge software (Fujifilm Co.). Protein levels were evaluated using the Image Gauge software, and image processing (resizing, cropping, and merging) was conducted using Adobe Photoshop (Adobe Systems).

### 2.6 ELISA

Ten thousand mouse aAVCs were co-cultured with 1 × 10^5^ NKT hybridoma (1.B2) for 24 h in 96-well round-bottom plates. Culture supernatants were analyzed for mouse IL-2 production using ELISA for cytokines with a mouse IL-2 Duo set (R&D Systems).

### 2.7 Enzyme-linked immune absorbent spot assay

Murine IFN-γ-secreting CD8^+^ T cells were performed by ELISPOT assays as previously described (27). Ninety six well filtration plates were coated with rat anti-mouse IFN-γ (R4-6A2; BD) overnight at 10 µg/mL. CD8^+^ T cells were isolated from the spleens of naïve or aAVC-treated mice by using CD8 MACS beads (Miltenyi Biotec). To prepare for antigen-presenting cells (APCs), splenic DCs from naïve mice were isolated using CD11c MACS beads. Additionally, CD8^+^ T cells (5 × 10^5^/well) were co-cultured with DCs (1 × 10^5^/well) pulsed with or without PepTivator (Miltenyi Biotec) for 24 h. After the culture, the plates were incubated with biotinylated anti-mouse IFN-γ (XMG1.2; BD) (2 µg/mL) for 2 h. Finally, IFN-γ-secreting spots were developed with streptavidin-HRP (BD) and stable DAB substrate (Research Genetics). IFN-γ SFCs were quantified using microscopy.

### 2.8 Prophylactic and therapeutic antitumor effect of aAVCs in mice

For the prophylactic model for each antigen, female C57BL/6 mice were immunized intravenously with 5 × 10^5^ aAVC expressing the relevant antigen and inoculated subcutaneously with 1 × 10^5^ B16 cells expressing the relevant antigen after two weeks. For the therapeutic model, C57BL/6 mice were injected subcutaneously with 5 × 10^5^ tumor cells and treated with 5 × 10^5^ aAVC when tumor size reached around 50mm^3^. Tumor diameters were measured using a Vernier caliper every 2–3 days, and the tumor volume was calculated using the following equation: V = L × W2 × 0.52, where V is the volume, L is the length, and W is the width. Mice with high tumor burden (volume > 2000 mm^3^) were sacrificed under anesthesia.

### 2.9 Statistical analysis

StatMate V software (Nihon 3 B Scientific Inc., Japan) was used for the statistical analyses. A student’s *t*-test (two-tailed) was used for the analysis of differences between the two groups. One-way analysis of variance (ANOVA) and *post-hoc* Dunnett’s tests were used for multiple comparisons. *P* values less than 0.05 were considered significant (*P<0.05; **P<0.01; ***P<0.001).

## 3 Results

### 3.1 Establishment of prostate antigen-expressing aAVCs

**Figure 1:**
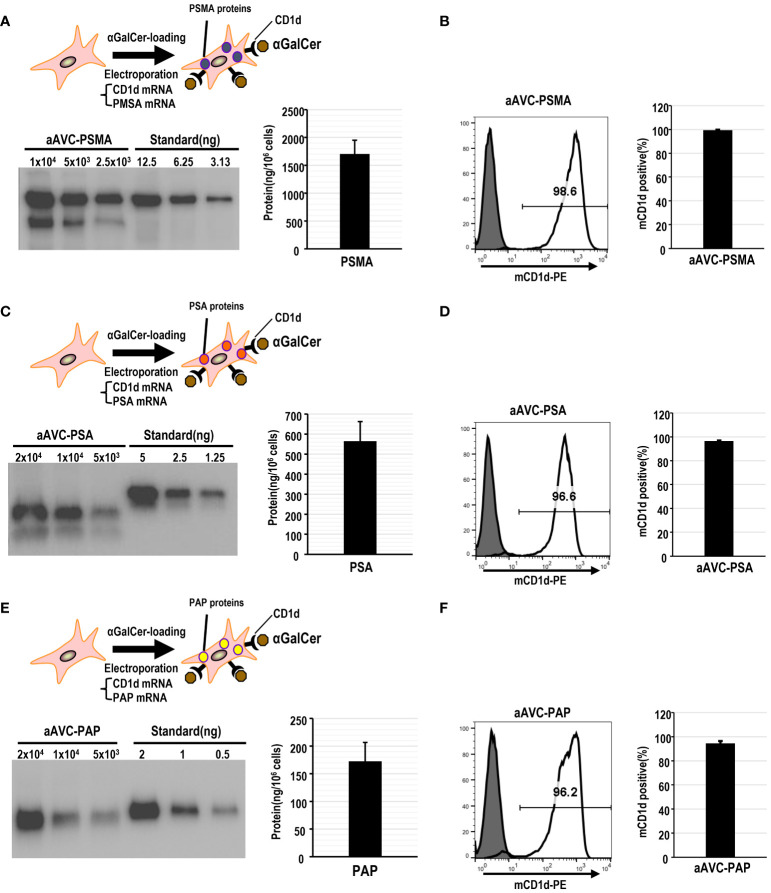
Establishment of monovalent aAVCs co-expressing CD1d and prostate tumor antigens for cancer therapy. **(A)** aAVCs expressing PSMA were created by electroporating NIH3T3 cells with PSMA and murine CD1d mRNA, followed by loading with α-galactosylceramide (α-GalCer). PSMA protein expression level was assessed using western blotting. **(B)** CD1d surface expression on aAVC-PSMA was assessed by flow cytometry (open, aAVC-PSMA; shaded, isotype). **(C)** aAVC-PSA was prepared as described in **(A)**, with PSA being transfected instead. PSA protein expression in aAVC-PSA was measured using western blot analysis. **(D)** CD1d surface expression on aAVC-PSA was assessed using flow cytometry (open, aAVC-PSA; shaded, isotype). **(E)** aAVC-PAP was prepared as described in **(A)**, with PAP transfected instead. The PAP protein in aAVC-PAP was measured using western blot analysis. **(F)** as similar to **(A)**, but CD1d surface expression on aAVC-PAP was assessed by flow cytometry (open, aAVC-PAP; shaded, isotype).

Mouse aAVCs were created by co-transfecting α-GalCer-loaded, mCD1d mRNA NIH3T3 cells with three human prostate antigen mRNAs (PSMA-mRNA, PSA-mRNA, or PAP-mRNA) and denoted as aAVC-PSMA (**Figures 1A, B**), aAVC-PSA (**Figures 1C, D**), and aAVC-PAP (**Figures 1E, F**), respectively. After transfection, we verified the expression of each protein using western blotting (**Figures 1A, C, E**). aAVC-PSMA, aAVC-PSA, and aAVC-PAP expressed the corresponding proteins at 1546 ± 202, 564 ± 97, and 172 ± 34 ng/10^6^ cells, respectively. We verified that the expression rate of mCD1d in each aAVC was > 90% (**Figures 1B, D, F**).

### 3.2 iNKT cell activation by prostate antigen-expressing aAVCs

**Figure 2:**
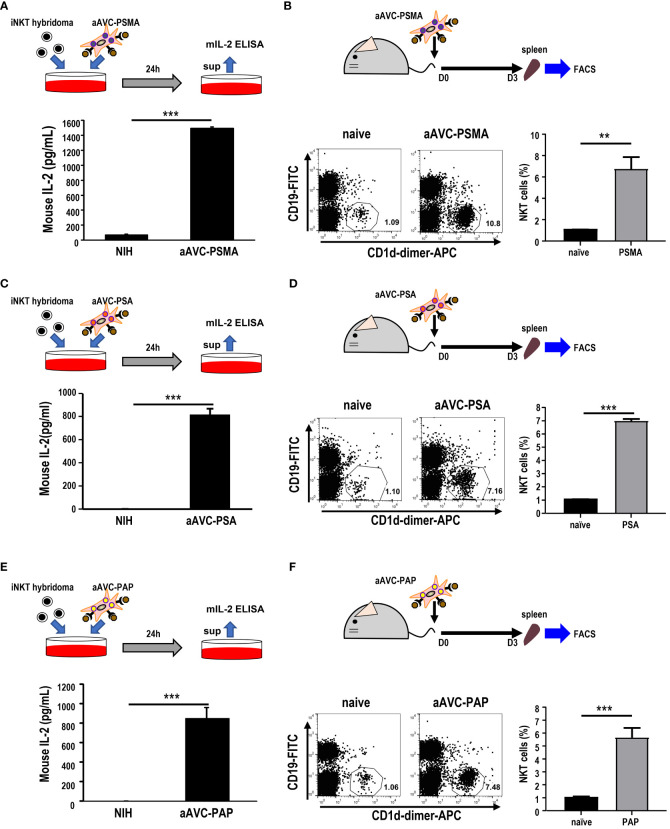
iNKT cell activation by aAVCs. **(A, C, E)** α-GalCer presentation on aAVC-PSMA **(A)**, aAVC-PSA **(B)**, and aAVC-PAP **(C)**. aAVCs were co-cultured with the Vα14 iNKT cell hybridoma 1.B2 for 24 h (upper), and IL-2 production in the culture supernatant was evaluated using IL-2 ELISA (n=4, mean ± SEM) (lower); ***P<0.001 (Mann-Whiteny). **(B, D, F)** C57BL/6 mice were injected intravenously with 5 × 10^5^ aAVC-PSMA **(B)**, aAVC-PSA **(D)**, and aAVC-PAP **(F)**. Spleens were removed after 3 days, and splenic iNKT was analyzed after staining with CD19-FITC, CD1d-dimer^+^ APC, and 7-AAD (upper). Representative dot plots showing the frequency of splenic iNKT cells (lower left) and summary(n=3, mean ± SEM) (lower right) were shown. **P<0.01, ***P<0.001(Mann-Whiteny).

After verifying the establishment of each aAVC type, we evaluated whether aAVC-PSMA, aAVC-PSA, and aAVC-PAP induced iNKT cell activation. We examined the iNKT response by culturing these aAVCs with iNKT cell hybridomas *in vitro*. After culturing aAVC-PSMA, aAVC-PSA, and aAVC-PAP with iNKT cells, IL-2 was produced by iNKT in the presence of aAVC, but not parental NIH cells in all groups (**Figures 2A, C, E**). Next, we investigated the capacity of each aAVC to expand iNKT cells *in vivo*. The percentage of iNKT cells in the spleen was examined three days after administering each aAVC. The iNKT cell frequency in all aAVC-PSMA-, aAVC-PSA-, and aAVC-PAP-injected mice was five times higher than that in the naïve mice (**Figures 2B, D, F**). Therefore, aAVC-PSMA, aAVC-PSA, and aAVC-PAP similarly stimulated iNKT cells *in vivo*, with aAVC-PSMA demonstrating the highest potency as an activator of iNKT cells in this context.

### 3.3 Prophylactic effects of aAVC-PSMA, aAVC-PSA, or aAVC-PAP administration on prostate antigens

**Figure 3:**
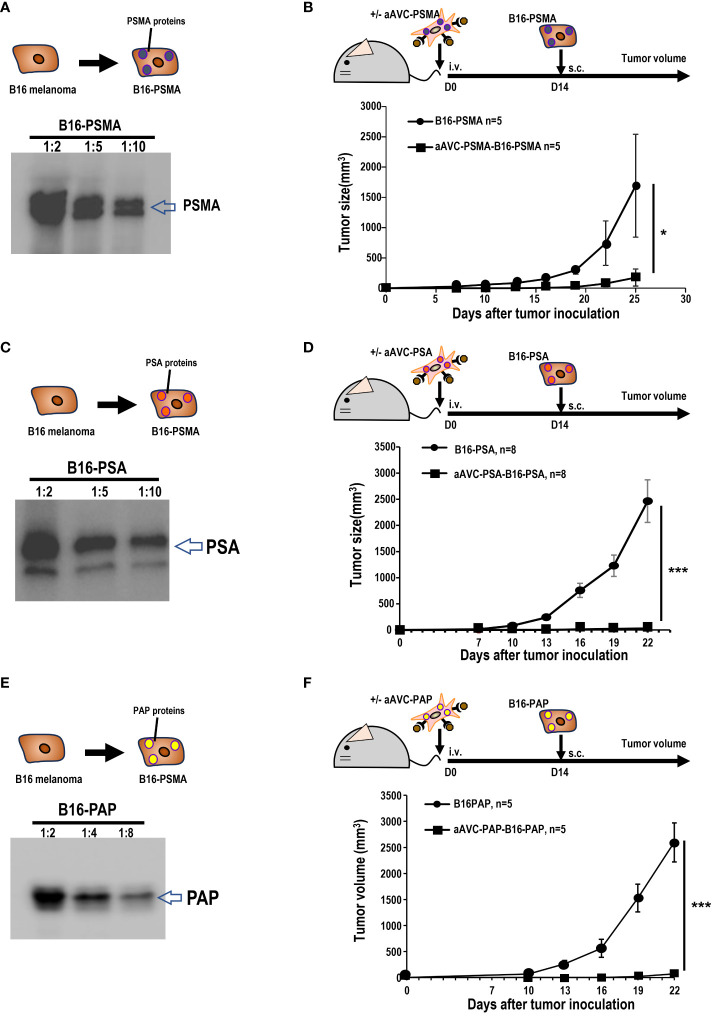
Antitumor response by aAVCs in a prophylactic model. **(A)** Establishment of B16-PSMA **(A)**, B16-PSA **(C)**, and B16-PAP **(E)** cells. After introducing the PSMA, PSA, or PAP gene into B16, stable PSMA, PSA, or PAP protein expression was verified using western blot analysis. **(B, D, F)** Mice were immunized with or without 5 × 10^5^ aAVC-PSMA, aAVC-PSA, or aAVC-PAP on day 0. Following this, mice were challenged with 5 × 10^5^ B16-PSMA **(B)**, B16-PSA **(D)**, or B16-PAP **(F)**, respectively on day 14 (n=5 per group, mean ± SEM); *P<0.05. ***P<0.001.

Owing to its resistance to ICB, B16 melanoma is widely recognized as a cold tumor. To evaluate whether the three types of prostate antigen-expressing aAVCs exhibit antitumor effects, we established three permanent B16 cell lines: B16 expressing PSMA, PSA, or PAP (**Figures 3A, C, E**). C57BL/6 mice were intravenously administered aAVC-PSMA, aAVC-PSA, or aAVC-PAP individually and then inoculated with PSMA-, PSA-, or PAP-expressing melanoma cell lines (B16-PSMA, B16-PSA, and B16-PAP) respectively after 14 days. Compared with the mice in the untreated control group, the aAVC-PSMA-, aAVC-PSA-, or aAVC-PAP-vaccinated mice were protected from the growth of B16-PSMA, B16-PSA, and B16-PAP (**Figures 3B, D, F**). Vaccination with monovalent prostate antigen-expressing aAVC exhibited substantial antitumor effects.

### 3.4 Relationship between iNKT frequency and antigen-specific T cell immunity in aAVC therapy

**Figure 4:**
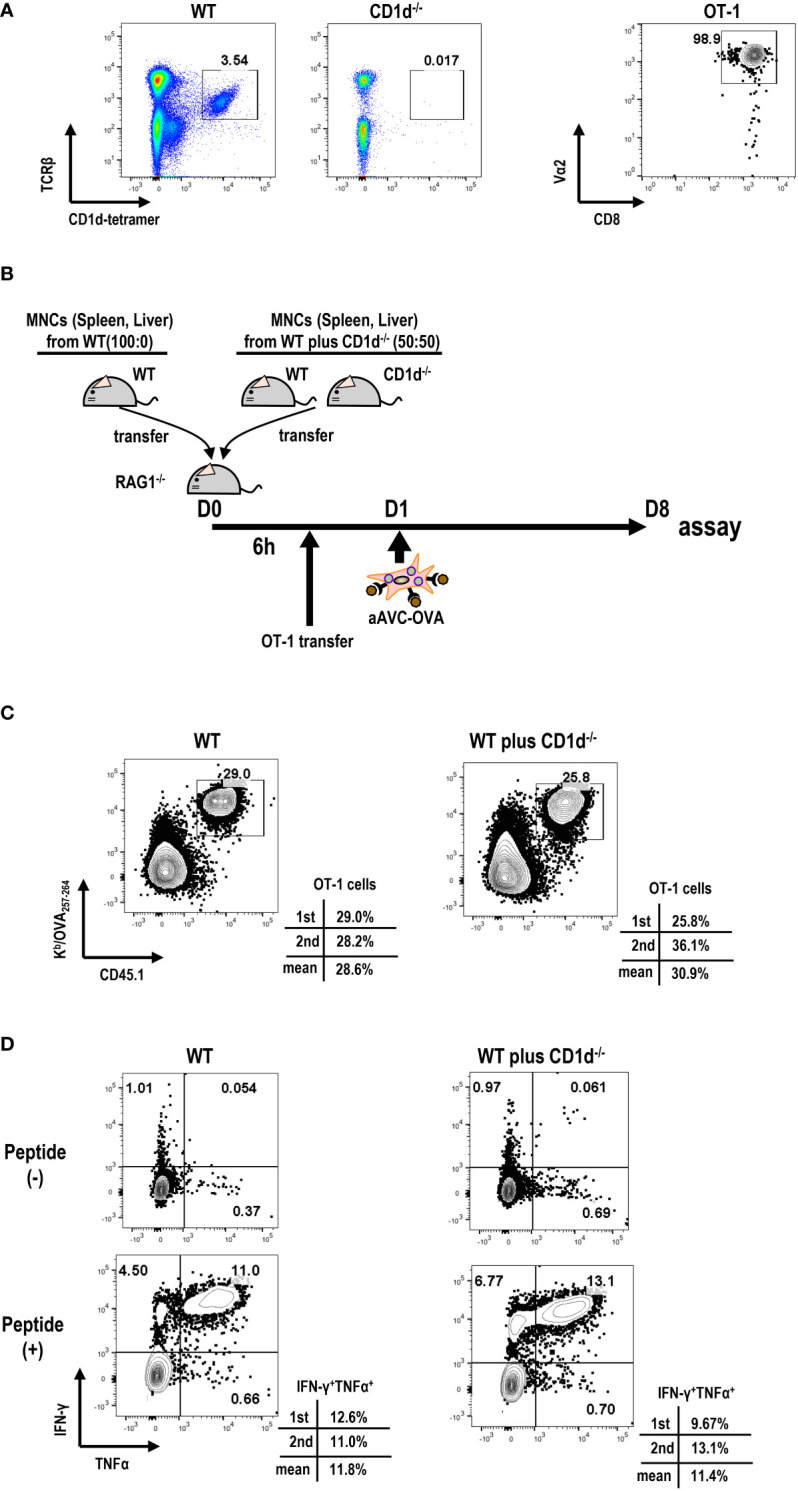
Relation of iNKT cells in host to antigen-specific T cell immunity by aAVC therapy. **(A)** Preparation of MNCs and OT-1 cells. Frequency of iNKT cells in B cell-depleted MNCs from WT and CD1d^-/-^ mice (right) and purity of OT-1 CD8^+^ T cells (left) were analyzed by flow cytometry. OT-1 CD8^+^ T cells were isolated from spleen and lymph nodes of Ly5.1 OT-1Tg mice using CD8 MACS beads. The purity of OT -1 cells was analyzed using CD8a-FITC and Va2-PE. **(B)** Experimental protocol. The MNCs from WT mice and CD1d^-/-^ mice (40x10^6^/mouse) at the ratio of 50:50 (%) or 100:0(%) were transferred to Rag1^-/-^ mice. OT-1 cells (1x10^5^/mouse) were transferred 6 h later. aAVC-OVA cells (5x10^5^/mouse) were administered the following day. **(C, D)** Frequency and cytokine production of OVA-specific CD8^+^ T cells. A week later, the Kb/SIINFEKL-tetramer^+^ CD8^+^T cells **(C)** and OVA257–264 peptide specific cytokine production (IFN-γ and TNF-α) **(D)** were analyzed. The data are representative data from two experiments independently and each data are also provided.

Next, we examined the relationship between iNKT cell frequency and antigen-specific T cell immunity in aAVC therapy. For this, we transferred different number of iNKT cells to Rag1^-/-^ mice. We harvested B cell-depleted MNCs from spleen and liver of WT mice and CD1d^-/-^ mice that are deficient in iNKT cells at the ratio of 100:0(%) or 50:50 (%) to Rag1^-/-^ mice (**Figures 4A, B**). We verified that the number of iNKT cells (1^st^; 0.74x 10^6^ cells/mouse, 2^nd^ 0.53 x 10^6^ cells/mouse) included in MNCs from WT mice and CD1d^-/-^ mice 50:50 (%) was half the number of iNKT cells included n MNCs from WT mice (1^st^; 1.41x 10^6^ cells/mouse, 2^nd^ 1.05x 10^6^ cells/mouse). Then, to trace and measure the antigen-specific T cell response, we administered low number of OT-1 cells (1x 10^5^ cells/mouse) and subsequently aAVC-OVA cells the following day. Despite the reduced frequency of iNKT cells, the OVA-specific CD8^+^ T cell response in mice given iNKT cells from WT mice and CD1d^-/-^ mice wassimilar to those from WT mice-derived iNKT cells transferred Rag1^-/-^ mice (**Figures 4C, D**). Thus, although the activation of iNKT cells is important factor, even if low frequent iNKT cells *in vivo* can show the adjuvant activity for T cell priming sufficiently in aAVC therapy.

### 3.5 Therapeutic effect of aAVC-PSMA, aAVC-PSA, or aAVC-PAP on prostate antigen-expressing tumor

**Figure 5:**
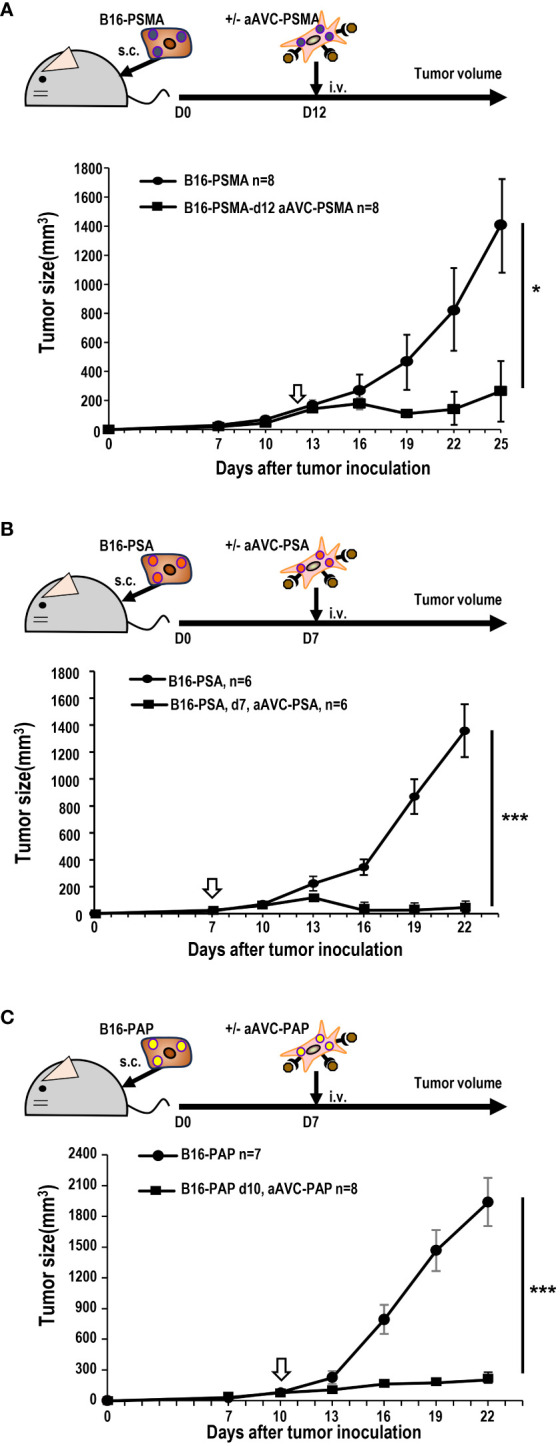
Antitumor response by aAVCs in a therapeutic model. The antitumor response was evaluated in a therapeutic model in which the therapy was administered at a tumor volume of around 50 mm^3^. **(A)** Mice were inoculated with 5 × 10^5^ B16-PSMA cells and treated with or without 5 × 10^5^ aAVC-PSMA on day 12 (n=8 per group, mean ± SEM); *P<0.05. **(B)** Mice were inoculated with 5 × 10^5^ B16-PSA cells and treated with or without 5 × 10^5^ aAVC-PSA on day 7 (n=6 per group, mean ± SEM); ***P<0.001. **(C)** Mice were inoculated with 5 × 10^5^ B16-PAP cells and treated with or without 5 × 10^5^ aAVC-PAP on day 10 (n=7–8 per group, mean ± SEM); ***P<0.001.

Next, we investigated whether aAVC therapy had a therapeutic effect on prostate antigen-expressing tumor-bearing mice. B16-PSMA, B16-PSA, and B16-PAP cells were inoculated subcutaneously into mice individually. When we observed tumors measuring around 50 mm^3^ in all tumor-bearing mice, they were treated with aAVC-PSMA (**Figure 5A**), aAVC-PSA (**Figure 5B**), or aAVC-PAP (**Figure 5C**), respectively. Each aAVC exhibited tumor abrogation individually (**Figures 5A–C**). Among these, aAVC-PSA appears to have the most potent therapeutic effect.

### 3.6 Demonstration of tumor epitope spreading by aAVC therapy

**Figure 6:**
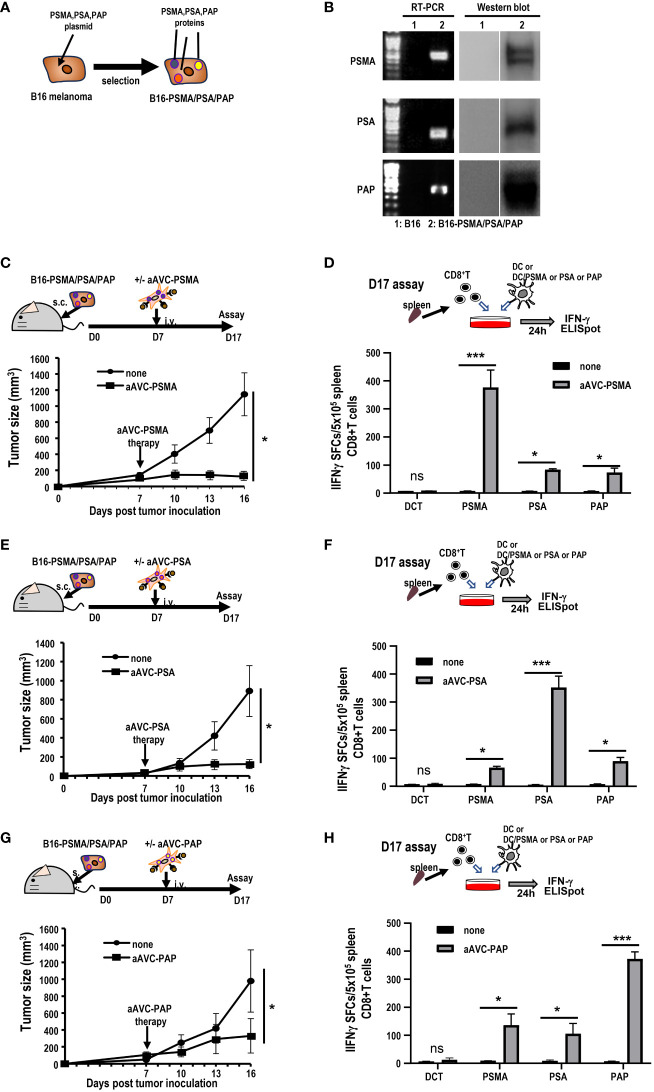
Induction of tumor epitope spreading by aAVC vaccination. **(A, B)** B16-PSMA/PSA/PAP cells were established using a retroviral vector expressing PSMA, PSA, or PAP, respectively. Tumor expression was verified using RT-PCR and western blotting. **(C, E, G)** Antitumor effect of various prostate antigen-expressing aAVC therapies. Mice were inoculated with 5 × 10^5^ B16-PSMA/PSA/PAP cells and treated with or without 5 × 10^5^ aAVC-PSMA **(C)**, aAVC-PSA **(E)**, or aAVC-PAP **(G)** on day 7 (n=5 per group, mean ± SEM); *P<0.05. **(D, F, H)** Epitope spread of prostate antigens in CD8^+^ T cells after aAVC therapy. Ten days after vaccination with aAVC-PSMA **(B)**, aAVC-PSA **(D)**, or aAVC-PAP **(F)**, PSMA-, PSA-, or PAP-specific CD8^+^ T cells were analyzed using an IFN-γ ELISPOT assay. For this, splenic CD8^+^ T cells isolated from the immunized mice were cultured with splenic CD11c^+^ dendritic cells (DCs) from naïve mice that had been cultured in the presence or absence of PSMA, PSA, or PAP-PepTivator for 24 h. (n=4 per group, mean ± SEM); *P<0.05; ***P<0.001; *ns*: not significant, according to Tukey’s test.

Next, we investigated whether aAVC therapy induces antigen spreading in a therapeutic model. We established three antigen-expressing tumor cell lines by introducing DNA of PSMA, PSA, and PAP into B16 cells (hereafter, B16-PSMA/PSA/PAP) (**Figure 6A**). We verified that these cells expressed each antigen using RT-PCR and western blot analyses (**Figure 6B**). B16-PSMA/PSA/PAP-bearing mice were treated with monovalent prostate cancer antigen expressing aAVC, that is, either aAVC-PSMA, aAVC-PSA, or aAVC-PAP on day 7. We found that immunization with aAVC-PSMA, aAVC-PSA, or aAVC-PAP abrogated the tumors (**Figures 6C, E, G**). After verifying the antitumor effect on day 16, we sacrificed the mice. We analyzed the antigen-specific CD8^+^ T cell response: PSMA-specific T cell response in aAVC-PSMA-treated mice (**Figure 6D**), PSA-specific T cell response in aAVC-PSA-treated mice (**Figure 6F**), and PAP-specific T cell response in aAVC-PAP-treated mice (**Figure 6H**). We detected a higher antigen-specific CD8^+^ T cell response following treatment with the relevant antigen-expressing aAVC. We found the antitumor effects through the therapy with aAVC-PSMA, aAVC-PSA or aAVC-PAP respectively (**Figures 6C, E, G**). In addition, intriguingly, we found that monovalent-aAVC induced not only CD8^+^ T cell responses against the target antigen, but also elicited antigen-specific CD8^+^ T cell responses against other tumor antigens simultaneously. This discrepancy might be explained by the capacity of aAVC therapy to induce significant epitope spreading from the tumor to CD8^+^ T cells.

### 3.7 Multiple T cell responses induced by treatment with a multivalent antigen-expressing aAVC

**Figure 7:**
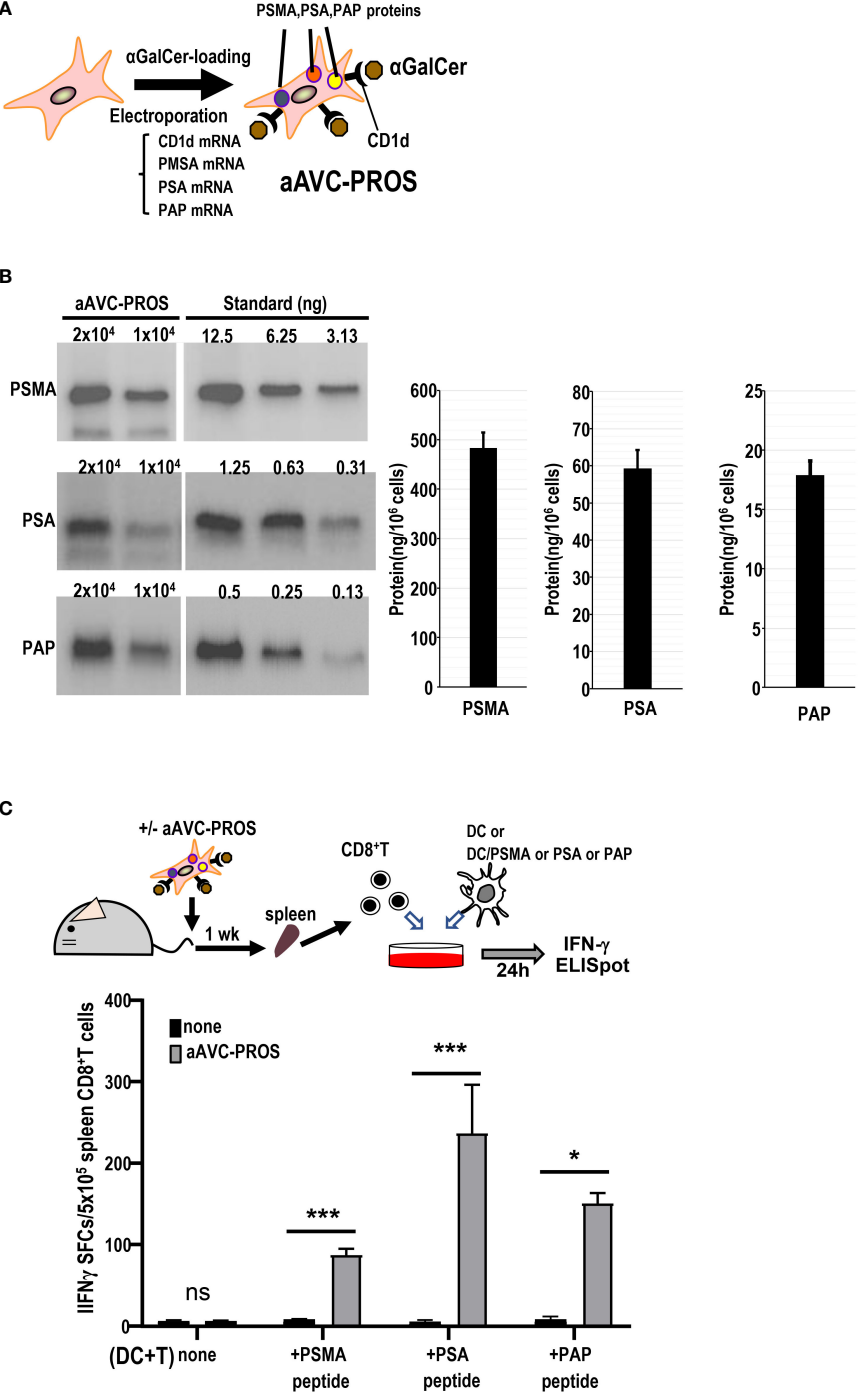
Establishment of a multivalent aAVC and prostate cancer antigen-specific CD8^+^ T cell response. **(A)** Establishment of multivalent artificial adjuvant vector cells for the prostate (aAVC-PROS). Three prostate cancer antigen-expressing artificial adjuvant cells (aAVC-PSMA/PSA/PAP and aAVC-PROS) were established by co-transfecting NIH3T3 cells with PSMA, PSA, PAP, and murine CD1d mRNA and then loaded with α-GalCer. **(B)** Tumor protein antigen in aAVC-PROS. The amount of PSMA, PSA, and PAP was determined using western blot analysis. **(C)** The prostate antigen-specific CD8^+^ T cell response induced by aAVC. Mice were intravenously immunized with 5 × 10^5^ aAVC-PROS. After one week, PSMA-, PSA-, or PAP-specific CD8^+^ T cells were analyzed using an IFN-γ ELISPOT assay. Splenic CD8^+^ T cells isolated from the immunized mice were cultured with splenic CD11c^+^ DCs from naïve mice that had been cultured in the presence or absence of PSMA-, PSA-, or PAP-PepTivator for 24 h. (n=4 per group, mean ± SEM) *P<0.05; ***P<0.001; *ns*: not significant, according to Tukey’s test.

Subsequently, we generated a multivalent aAVC expressing the three prostate cancer antigens (**Figure 7A**). After simultaneous transfection with PSMA, PSA, and PAP mRNA in addition to murine CD1d mRNA, we verified the expression of PSMA, PSA, and PAP protein by western blot analysis (**Figure 7B**). The trivalent cancer antigen aAVC, denoted as aAVC-PROS, effectively expressed PSMA (483 ± 31ng/10^6^ cells), PSA (59.2 ± 5.0 ng/10^6^ cells), and PAP (17.9 ± 1.2 ng/10^6^ cells). CD8^+^ T cells were isolated from the spleen cells of vaccinated mice that had been administered aAVC-triPROS to analyze the antigen-specific T cell response. At day 7, they were cocultured with splenic DCs from naïve mice in the presence or absence of PSMA, PSA or PAP peptivators. The antigen-specific CD8^+^ T cells produced in response to the peptides were determined using the ELISpot assay (**Figure 7C**). Although the amounts of each protein expressed in aAVC-PROS were different, the T cell response induced by aAVC-PROS was higher in PSA-specific T cells, followed in order by PAP-specific T cells and PSMA-specific T cells. The amount of protein expressed in the aAVC did not always correlate with the T cell immune response. T cell immune response depends on the amount of antigen present and antigenicity of the specific expressed protein. Our results revealed that the response depends on the antigen quantity when the antigens are identical. However, when dealing with different antigens, the response relies more on antigenicity than the amount of antigen present in the aAVCs. This suggests that the T cell response may depend on the immunogenicity of the antigen involved in aAVC when we use multifunctional epitope-expressing aAVC.

### 3.8 Therapeutic effect of divalent and trivalent antigen-expressing aAVCs

**Figure 8:**
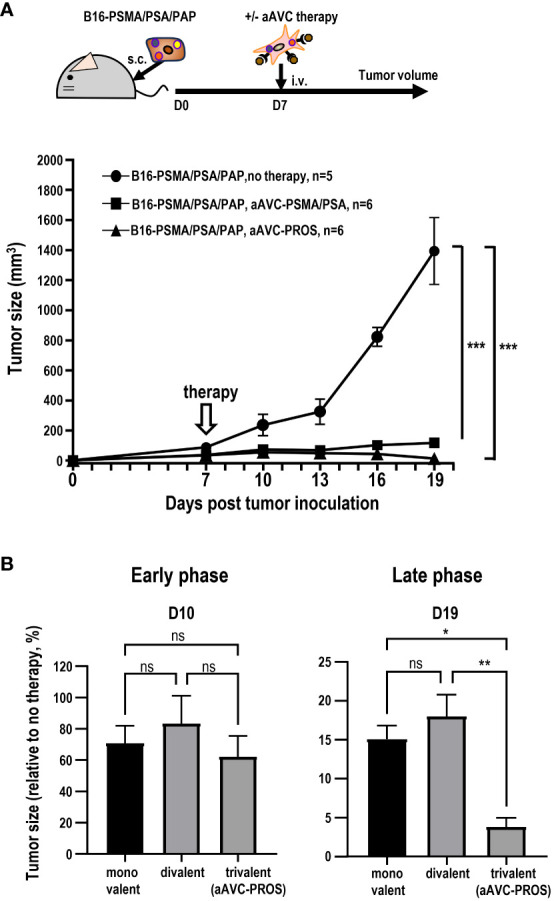
Comparison of the antitumor effect of divalent and trivalent prostate antigen-expressing aAVCs. Divalent artificial adjuvant cells (aAVC-PSMA/PSA) were established by co-transfecting NIH3T3 cells with PSMA, PSA, and murine CD1d mRNA. **(A)** Mice were inoculated with 5 × 10^5^ B16-PSMA/PSA/PAP cells and treated with or without 5 × 10^5^ divalent aAVC-PSMA/PSA or trivalent aAVC-PROS on day 7. **(B)** Comparison of the antitumor effects of monovalent (aAVC-PSMA, aAVC-PSA, or aAVC-PAP), divalent (aAVC-PSMA/PSA), or trivalent aAVCs (aAVC-PROS). The tumor size was evaluated by comparing untreated mice with treated mice on days 10 (Early phase) and 19 (Late phase). *Plt;0.05, **P<0.01, ***P<0.001; ns: Tukey’s test.

Having determined that monovalent antigen-expressing aAVC therapy is effective against multiple antigen-expressing tumors and that this effect was caused by epitope spreading, we subsequently investigated whether divalent or trivalent antigen-expressing aAVC generated antitumor effects more effectively. Because PSMA and PSA were more effective than PAP, suggesting that combining these two antigens would be more potent (**Figure 6**), we established a divalent aAVC expressing PSMA and PSA. We compared the antitumor effects of divalent aAVC-PSMA/PSA and trivalent aAVC-PROS against B16-PSMA/PSA/PAP (**Figure 8A**). Both vaccinations showed an abrogation of tumor cells similarly. Further analysis indicates that divalent aAVC is better than monovalent aAVC; however, there was no difference in efficacy between divalent and trivalent aAVCs. Finally, we compared the antitumor effects of the three vaccines during the early and late tumor phases. When we assessed the tumor size on days 10 (early) and 19 (late), we did not observe any difference between divalent and trivalent aAVC vaccination until day 10, but found that the antitumor effect of trivalent aAVC was superior to that of monovalent or divalent aAVC on day 19 (**Figure 8B**). These findings imply that even monovalent aAVC show the antitumor effect sufficiently during the early phase. Notably, only trivalent antigen-expressing aAVC exhibits increased potency in the late phase. Thus, multivalent antigen-expressing aAVC can elicit multiple T cell responses more effectively than monovalent antigen-expressing aAVC for the long period.

## 4 Discussion

In this study, we established an aAVC approach using three prostate cancer antigens as aAVC-PROS and verified the advantages of this vaccine in a cold tumor melanoma model. Differences in the immune response and antitumor effects were evaluated for each antigen. T-cell responsiveness differs depending on the tumor antigen characteristics. Essentially, the strength of the CD8^+^ T cell response depends on the antigenicity and quantity of tumor antigen in the aAVC. Therefore, we first compared the capacity of aAVCs expressing three different antigens to activate innate and adaptive immunity. The results showed that iNKT responses, antigen-specific CD8^+^ T cells, and antitumor activity were successfully induced by all aAVCs. We also found that if the antigen in each aAVC were the same one, ensuing T cell response would depend on the amount of antigen, however, if the antigen in aAVC was different, it would depend on the antigenicity. Multivalent vaccines are regarded as an effective strategy to prevent immune escape in cases where one type of tumor antigens is lost. In the current study, we verified that aAVC-PROS carrying three antigens exhibited sufficient antitumor effects and induced three antigen-specific CD8^+^ T cells. Interestingly, when tumors expressing multiple antigens were treated with monovalent aAVCs, CD8^+^ T cells were elicited against antigens that differed from those with which the mice were immunized. Thus, we demonstrate that aAVC induces epitope spreading. Finally, we evaluated the direct activity of multivalent antigen-expressing aAVC in conjunction with the indirect activity of tumor epitope spreading by aAVC by comparing monovalent, divalent, or trivalent antigen-expressing aAVC. In the early phase, there was a minimal difference among the vaccines; however, multivalent antigen-expressing aAVC exhibited more potent antitumor effects in the late phase. Our results suggest that antigen-spreading ability and multivalent antigen vaccines are beneficial for long term-antitumor effects. aAVCs targeting multiple prostate cancer antigens would be promising therapeutic options for conventional therapy-resistant prostate cancer.

Epitope spreading was first described in experimental autoimmune encephalomyelitis (39). These antigens often segregate into different epitopes derived from defined proteins (intramolecular spreading) and other antigens (intermolecular spreading). Antigen spread following treatment with sipuleucel-T has been demonstrated most clearly through studies of antibody responses in patients with prostate cancer in a phase III trial (40). Evidence of antigen spread in response to immunotherapy has also been reported in several other tumor types, including metastatic breast cancer (41) and melanomas (42–44). Moreover, emerging data have revealed that patients who acquire broad immunological responses have improved clinical outcomes (41, 45, 46). Although it is theoretically understood that epitope spreading is induced, it cannot be simply and easily induced using radiation, chemotherapy drugs, or ICB. Therefore, it is essential to determine whether the immune response can adapt to amplify and diversify the endogenous T cell response against diverse tumor antigens. This clarification is crucial for understanding the potential of immune responses against multiple tumor antigens. Epitope spreading and endogenous T cell activation within the tumor can be promoted by cDC1 in the tumor (47). Since DCs *in vivo* seem to play a critical role in the success of epitope spreading in the antitumor effect, they could serve as a surrogate molecular indicator of the clinical impact of the aAVC platform.

According to concerns for patients who have the low frequency of iNKT cells, several groups demonstrated that NK cell response was shown after α-GalCer-pulsed DC therapy (24, 26, 37, 48). Particularly, an initial study showed that NK cell response depends on the function of iNKT cells, but not the frequency. We observed a notable granzyme B-expressing NK cell response in most lung cancer patients undergoing autologous DC/Gal therapy (26). However, we did not observe a similar response in T cells. Recently, we conducted a phase I trial involving aAVC-expressing WT1 therapy targeted at patients with relapsed and refractory AML (37). Our results revealed that either iNKT or NK cells or both were activated in all the patients, even in patients with lung cancer and relapse and refractory AML, including those with low counts of iNKT cells. We demonstrated that a small number of iNKT cells exert an adjuvant effect in murine model (**Figure 4**). Our future research will explore whether an increased number of transferred iNKT cells can enhance T cell functionality more effectively.

Several strategies have been explored to improve the efficacy of immunotherapy, including monotherapy, multivalent therapy, and combination therapies. In the present study, we compared monovalent and multivalent (divalent or trivalent) antigen-expressing aAVCs. We demonstrated that multivalent antigen-expressing aAVC was more effective than monovalent-aAVC. When we subsequently compared divalent or trivalent aAVC, we revealed that trivalent antigen-expressing aAVCs enhance antigen-specific T cells under tumor-bearing, immunosuppressive conditions. When considering combination therapy for prostate cancer, it is crucial to consider amplifying antigen-specific CD8^+^ T cells, efficiently trafficking them by TIME reprogramming, and reinvigorating and sustaining their survival via ICB and cytokines. A recent study investigating ICB resistance in prostate cancer showed that androgens worsen the response to ICB (49). Androgen receptor (AR) blockade directly enhances the PSA antigen-specific CD8^+^ T cell response and restores the activation of pathways in CD8^+^ T cells, including TCR, PD-1, and IFN-γ signaling (49). The enhancement of these pathways corresponds to the deactivation of the NR4A1 pathway, which limits the efficacy of anti-PD-1 antibody-targeted immunotherapy (50). We previously demonstrated that the two-fold T cell response in the TIME was elevated by the synergistic effect of aAVC and anti-PD1Ab on T cells in the TIME (29), indicating its potential application in prostate cancer. Thus, analysis of the TIME may reveal various mechanisms for effective checkpoint blockade in tumor-bearing hosts, leading to the development of new therapeutic strategies.

## Data availability statement

The original contributions presented in the study are included in the article. Further inquiries can be directed to the corresponding author.

## Ethics statement

The animal study was approved by Institutional Animal Care Committee of RIKEN. The study was conducted in accordance with the local legislation and institutional requirements.

## Author contributions

SY: Formal Analysis, Investigation, Methodology, Visualization, Writing – original draft. KS: Formal Analysis, Investigation, Methodology, Validation, Writing – original draft. SF: Conceptualization, Formal Analysis, Funding acquisition, Methodology, Supervision, Validation, Writing – original draft.
